# 69399 How are substance use disorder treatment programs in Arkansas responding to COVID-19? A qualitative study

**DOI:** 10.1017/cts.2021.550

**Published:** 2021-03-30

**Authors:** Jure Baloh, Geoffrey M. Curran

**Affiliations:** 1 University of Arkansas for Medical Sciences; 2 University of Arkansas for Medical Sciences, Central Arkansas Veterans Healthcare System

## Abstract

ABSTRACT IMPACT: This study informs how substance use treatment programs responded to the COVID-19 pandemic, and highlights implication for future translational research and practice. OBJECTIVES/GOALS: The COVID-19 pandemic rapidly changed how substance use disorder (SUD) treatment services are organized and provided. This study examined what changes SUD treatment programs in Arkansas implemented (e.g., guidelines, technologies), and what factors influenced their ability to implement and sustain these changes. METHODS/STUDY POPULATION: Between May and August 2020, we conducted semi-structured phone interviews with 29 leaders (administrative and/or clinical leaders) at 21 residential and outpatient SUD treatment programs throughout Arkansas (i.e., in all five Arkansas public health regions). Interviews were based on the Consolidated Framework for Implementation Research and focused on what changes programs were implementing in response to the COVID-19 pandemic, barriers and facilitators to implementation, and recommendations for future. The interviews were on average about 30 minutes long, and we provided no participant compensation. Interviews were recorded and transcribed verbatim, then thematically analyzed. RESULTS/ANTICIPATED RESULTS: Programs implemented similar infection control practices: screening at entry, masks, hand hygiene, and social distancing. Residential programs stopped outside visitations and some capped admissions; outpatient programs stopped group sessions and switched most services to telehealth. Key facilitators included grants/loans (e.g., salaries), looser regulatory restrictions (e.g., telehealth), and good coordination with other organizations (e.g., state agencies). Key barriers included limited access to supplies (e.g., masks), no rapid testing (particularly for residential care), limited capacity for social distancing, and negative employee and client responses (e.g., anxiety). Key recommendations include better access to supplies and testing, telehealth continuation and better communication. DISCUSSION/SIGNIFICANCE OF FINDINGS: This study provides an insight into how SUD programs responded to the COVID-19 pandemic and what the ‘new normal’ is. This can inform D&I studies conducted in SUD settings, including studies examining what implementation strategies can help sustain these changes, or studies of other practices implemented during or after the pandemic.

